# SynDIG1 Promotes Excitatory Synaptogenesis Independent of AMPA Receptor Trafficking and Biophysical Regulation

**DOI:** 10.1371/journal.pone.0066171

**Published:** 2013-06-13

**Authors:** Kathryn L. Lovero, Sabine M. Blankenship, Yun Shi, Roger A. Nicoll

**Affiliations:** 1 Departments of Cellular and Molecular Pharmacology and Physiology, University of California San Francisco, San Francisco, California, United States of America; 2 Neuroscience Graduate Program, University of California San Francisco, San Francisco, California, United States of America; Institute for Interdisciplinary Neuroscience, France

## Abstract

AMPA receptors–mediators of fast, excitatory transmission and synaptic plasticity in the brain–achieve great functional diversity through interaction with different auxiliary subunits, which alter both the trafficking and biophysical properties of these receptors. In the past several years an abundance of new AMPA receptor auxiliary subunits have been identified, adding astounding variety to the proteins known to directly bind and modulate AMPA receptors. SynDIG1 was recently identified as a novel AMPA receptor interacting protein that directly binds to the AMPA receptor subunit GluA2 in heterologous cells. Functionally, SynDIG1 was found to regulate the strength and density of AMPA receptor containing synapses in hippocampal neurons, though the way in which SynDIG1 exerts these effects remains unknown. Here, we aimed to determine if SynDIG1 acts as a traditional auxiliary subunit, directly regulating the function and localization of AMPA receptors in the rat hippocampus. We find that, unlike any of the previously characterized AMPA receptor auxiliary subunits, SynDIG1 expression does not impact AMPA receptor gating, pharmacology, or surface trafficking. Rather, we show that SynDIG1 regulates the number of functional excitatory synapses, altering both AMPA and NMDA receptor mediated transmission. Our findings suggest that SynDIG1 is not a typical auxiliary subunit to AMPA receptors, but instead is a protein critical to excitatory synaptogenesis.

## Introduction

The AMPA-type ionotropic glutamate receptors (AMPARs) underlie fast, excitatory synaptic transmission and plasticity in the brain [1] [2]. For years, the functional diversity of these tetrameric receptors was thought to originate solely from their subunit composition, which confer different biophysical properties and roles in synaptic transmission [3] [4]. Over the last decade, however, it has become clear that AMPAR function is also dependent on a multitude of interacting proteins, termed auxiliary subunits. AMPAR auxiliary subunits are typically defined as transmembrane proteins that bind directly to AMPARs, and, similar to other ion channel auxiliary subunits, alter ER trafficking, surface localization, subcellular targeting, and modulation of receptor biophysical properties [5] [6] [7] [8]. Studies of the different known AMPAR auxiliary subunits–including TARPs, CNIHs, CKAMP44, and GSG1L–have begun to elucidate the varying impact each has on AMPAR function and localization [9], [10] [11] [12] [13] [14] [15] [16], contributing greatly to our understanding of the diverse functional roles of AMPARs in the brain.

Recently, Synapse Differentiation Induced Gene 1 (SynDIG1) was identified as an AMPAR-interacting protein that regulates synaptic AMPAR content [17]. A type II transmembrane protein, its extracellular c-terminus was shown to bind directly to the AMPAR subunit GluA2 in COS-7 cells. Overexpression of SynDIG1 in dissociated hippocampal neurons led to a dramatic increase in miniature excitatory postsynaptic current (mEPSC) amplitude and frequency, along with increases in the density and size of AMPAR-containing synaptic puncta. shRNA-mediated knockdown of SynDIG1 had the opposite effect, greatly reducing mEPSC frequency and amplitude, while also decreasing the density and size of AMPAR-containing synaptic puncta. Yet, the mechanism by which changing SynDIG1 levels altered synaptic AMPAR-mediated transmission remains unstudied. Imaging of dissociated neurons showed a larger percentage of SynDIG1 colocalized with AMPARs at extrasynaptic than synaptic sites, and SynDIG1 expression levels positively correlated with surface AMPAR labeling [17], implying that SynDIG1 may regulate the surface trafficking of AMPARs. Additionally, owing to its binding to AMPARs, it is also possible that SynDIG1 alters AMPAR-mediated synaptic transmission by direct modification of channel gating properties.

In this study, we set out to further characterize the effect of SynDIG1 on excitatory transmission and determine if SynDIG1 acts as an auxiliary subunit of AMPARs. Through a battery of electrophysiological measurements, we show that SynDIG1 has no direct effect on AMPAR gating properties modulated by known auxiliary subunit interaction–including ligand binding affinity, deactivation, desensitization, and rectification–nor does SynDIG1 alter the surface trafficking of AMPARs. Instead, using hippocampal slice cultures, we make the surprising finding that in addition to regulating synaptic AMPARs, SynDIG1 also regulates NMDA receptor (NMDAR)-mediated transmission. We go on to show that SynDIG1 expression levels control the number of functional excitatory synapses in the hippocampus. Thus, we conclude that SynDIG1 does not act as a typical auxiliary subunit of AMPARs, but rather is a regulatory protein for excitatory synaptogenesis.

## Materials and Methods

### Ethics Statement

Animals were cared for in strict accordance with the UCSF Institutional Care and Use Committee guidelines. The protocol was approved by the UCSF IACUC, permit #AN085622-03.

### Molecular Biology

The SynDIG1 sequence from mouse (Accession number BC147352) was purchased from Open Biosystems, amplified from the pCR4-TOPO vector, and inserted into pIRES2-EGFP (Clontech) for expression in HEK cells and neurons. To knockdown SynDIG1 expression, we used an shRNA sequence (GCTGTGGCCAAAGGAGAC) that was previously verified [17] cloned into the FUGW vector (Addgene). Initially the shRNA oligo was cloned into pSuper (Oligoengine) and then transferred into the FHUGW vector (K. Roche). RNAi proof SynDIG1, containing three point mutations in the target region (GCCGTGGCCAAGGGGGAC), was cloned into pIRES2-DsRed to enable detection of both shRNA (EGFP) and RNAi proof target (DsRed) expression. To knockdown CNIH-2, we used an shRNA target sequence (GATGCGGTCTCTATCATGA), shown to be highly effective in reducing CNIH-2 protein levels [18], cloned into the FHUGW vector (H1-shRNA-pUb-EGFP).

### HEK Cell Electrophysiology

HEK cells (American Type Cell Culture) were used for expression of GluA1(Q)flip, GluA2(Q)flip, SynDIG1 and TARP γ-2. The cells were cultured in a 37°C incubator supplied with 5% CO_2_ and maintained in Dulbecco’s Modified Eagle Medium (DMEM, Invitrogen) supplemented with 10% fetal bovine serum. Transfection was performed using Lipofectamine2000 according to the manufacturer’s protocol (Invitrogen). For co-expressions, the ratio of GluA1 and GluA2 to SynDIG1 or TARP γ-2 cDNA was 1∶1. After transfection, cells were immediately dissociated with 0.05% trypsin and plated on coverslips pretreated with poly-D-lysine (BD Bioscience). To avoid cell death due to activation of exogenous glutamate receptors, the medium was supplemented with NBQX (50 µM, Tocris Bioscience). Recordings were made 1–2 days after transfection, using 4–5 MΩ glass electrodes filled with an internal solution consisting of 135 mM CsF, 33 mM CsOH, 2 mM MgCl_2_, 1 mM CaCl_2,_ 10 mM Hepes, 11 mM EGTA, and 0.1 mM spermine, pH 7.4. External perfusion medium consisted of 140 mM NaCl, 5 mM KCl, 5 mM EGTA, 1.4 mM MgSO_4_, 1 mM NaH_2_PO_4_, 10 mM glucose, and 10 mM Hepes, pH 7.2. 15 sec pulses of 1 mM glutamate (Abcam Biochemicals) or 1 mM kainate (Sigma) were applied–in the presence of 100 µM cyclothiazide (Abcam Biochemicals) to inhibit AMPAR desensitization–to record whole cell current amplitudes.

Current voltage relationships were recorded from outside-out patches under the same conditions used to obtain whole cell currents. Patches were held at −100 mV for 50 ms, then the voltage was ramped up to +100 mV at a rate of 2 mV/ms. Ramp sweeps were recorded before and during agonist application and at least 10 sweeps from each condition averaged. To subtract background leak conductance, the average ramp sweep before agonist application was subtracted from the average ramp sweep recorded during agonist application.

Fast responses to glutamate were recorded from outside-out patches using the following internal solution: 135 mM KF, 33 mM KOH, 2 mM MgCl_2_, 1 mM CaCl_2_, 11 mM EGTA, 10 mM Hepes, and 0.1 mM spermine, pH 7.2. Glutamate (1 mM) was dissolved in extracellular solution, along with 100 µM D-AP5 (Tocris Bioscience) and 500 nM tetrodotoxin (Tocris Bioscience) to isolate AMPAR-mediated currents. For deactivation experiments, 100 µM cyclothiazide (Abcam Biochemicals) was also included in the solution. 1 or 100 ms pulses of the glutamate solution were applied to patches every 5 s using a piezoelectric controller (Siskiyou). A single weighted decay calculated from the area under peak-normalized currents was used to measure AMPAR deactivation and desensitization, as described in a previous study [19]. Statistical significance was calculated using the Wilcoxon-Mann-Whitney test for unpaired data (Kaleidagraph).

### Slice Culture Electrophysiology

Hippocampal slice cultures were prepared from 6- to 8-day-old Sprague Dawley rats as described [20]. Transfections were carried out 1–3 days later with the Helios Gene Gun (Bio-Rad), using 1.0-µm gold particles (Bio-Rad) coated with 50 µg DNA.

Recordings were made 3–4 days (SynDIG1 overexpression) or 7–8 days (SynDIG1 shRNA) after transfection, using 3–4 MΩ glass electrodes filled with an internal solution consisting of 135 mM CsMeSO_4_, 8 mM NaCl, 10 mM Hepes, 4 mM Mg-ATP, 0.3 mM Na-GTP, 0.3 mM EGTA, 5 mM QX314-Cl, and 0.1 mM spermine, pH 7.2 with CsOH. External perfusion medium consisted of 119 mM NaCl, 2.5 mM KCl, 4 mM CaCl_2_, 4 mM MgSO_4_, 1 mM NaH_2_PO_4_, 26.2 mM NaHCO_3_, 11 mM glucose, 100 µM picrotoxin and 10 µM gabazine, saturated with 95% O_2_ and 5% CO_2_. In all experiments, transfected neurons–identified using fluorescence microscopy–and neighboring control neurons were recorded simultaneously. For synaptic recordings, a monopolar stimulating electrode was placed in stratum radiatum. A total of 30–50 stimulation trials were obtained at 0.2 Hz while holding the cells at −70 mV and +40 mV to record AMPAR-mediated and NMDAR-mediated currents, respectively. Series resistances typically ranged from 10 to 20 MΩ; a pair was discarded if the series resistances differed substantially between the two cells. Paired-pulse ratios were obtained by recording the responses to a pair of stimuli 40 ms apart, and quantified by measuring the peak amplitude of the second EPSC divided by the first. mEPSCs were obtained in the presence of 1 µM tetrodotoxin, with average amplitude and frequency being calculated from 200 synaptic events. Surface AMPAR EPSCs were elicited by local application of 500 nM S-AMPA (Abcam Biochemicals) and 50 µM cyclothiazide simultaneously to neighboring neurons for 0.5 ms. Sample size (n) for all evoked data refers to number of pairs. All paired-recording statistics were calculated using the Wilcoxon signed-rank test in Prism 5 (GraphPad); statistics for paired-pulse ratios were calculated using the Wilcoxon-Mann-Whitney test.

## Results

### SynDIG1 does not Influence Channel Gating or Surface Trafficking

**Figure 1:**
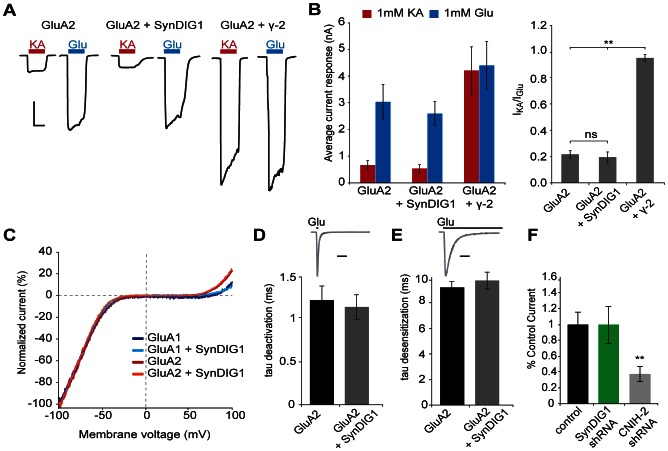
SynDIG1 does not alter biophysical properties or surface expression of AMPARs. (A) Representative whole cell currents recorded in response to application of 1 mM kainate (red) or 1 mM glutamate (blue) in HEK293T cells expressing GluA2(Q), GluA2(Q) and SynDIG1, or GluA2(Q) and TARP γ-2. Scale bar represents 10 s and 1000 pA. (B) Quantification of conditions shown in A showing the average current responses (left) and kainate to glutamate ratio (right) ± SEM. Note that while co-expression of the TARP γ-2 significantly increases kainate-induced current responses (n = 8, *p* = 0.0004), co-expression of SynDIG1 does not (n = 8, *p* = 0.91). (C) Normalized average current-voltage relationships for GluA1(Q)flip (n = 5), GluA1(Q)flip+SynDIG1 (n = 5), GluA2(Q)flip (n = 3), and GluA2(Q)flip+SynDIG1 (n = 3). The addition of SynDIG1 does not alter the current-voltage relationship in GluA1 (*p* = 0.94) or GluA2 (*p* = 0.83). (D) Scaled sample traces and average time course of deactivation recorded from HEK293T cells expressing GluA2(Q) (black, n = 10) or GluA2(Q)+SynDIG1 (gray, n = 6). Error bars represent SEM. No significant difference is found in the tau of deactivation after addition of SynDIG1 (*p* = 0.80). Scale bar represents 5 ms. (E) Scaled sample traces and average time course ± SEM of desensitization recorded from HEK293T cells expressing GluA2(Q) (black, n = 9) or GluA2(Q)+SynDIG1 (gray, n = 7). No significant difference is found in the tau of desensitization after addition of SynDIG1 (*p* = 0.38). Scale bar represents 10 ms. (F) Simultaneous whole cell currents recorded from wildtype, SynDIG1 shRNA expressing, and CNIH-2 shRNA expressing neurons recorded in response to application of 500 nM S-AMPA. Knockdown of the well-characterized AMPAR auxiliary subunit CNIH-2 leads to a dramatic decrease in surface responses (n = 6, *p* = 0.003), while no change in surface AMPARs is observed after knockdown of SynDIG1 (n = 7, *p* = 0.93). Bar graphs show mean response amplitude ± SEM.

Because SynDIG1 can bind directly to GluA2 [17], we questioned if SynDIG1 acts as an auxiliary subunit and alters AMPAR-mediated transmission by directly regulating biophysical properties and surface expression of AMPARs. We first examined the effect SynDIG1 expression has on AMPAR biophysical properties. Coexpression of the AMPAR auxiliary subunit TARP γ-2 with GluA2(Q)–the unedited form of the receptor that generates much larger currents–in HEK cells results in a highly significant increase in the response to kainate versus glutamate application, in agreement with previous findings that TARP association greatly increases kainate sensitivity of AMPARs [8]. In contrast, whole cell current responses to either glutamate or kainate application recorded from HEK cells expressing both GluA2 and SynDIG1 were indistinguishable from the responses of HEK cells expressing GluA2 alone (Figure 1A,B).

A signature of the TARP and CNIH auxiliary subunit interaction with AMPARs is a decrease in the block by intracellular polyamines at positive membrane potentials, detectable in the current-voltage relationship as reduced inward rectification [12] [21]. We recorded current-voltage relationships from outside-out patches obtained from HEK cells expressing GluA1 and GluA2 alone or in combination with SynDIG1 and found that SynDIG1 expression did not result in a change in AMPAR rectification, indicating that SynDIG1 expression does not affect intracellular polyamine affinity (Figure 1C).

Additionally, AMPAR-interacting proteins, such as TARPs and CKAMP44, have been shown to slow the deactivation time constant of AMPAR-mediated currents [11] [22] [23]. Yet, when we compared the deactivation rates after co-expression of SynDIG1 with GluA2, we observed no significant change in the deactivation time constant as compared to cells expressing GluA2 alone (Figure 1D). Similarly, when we measured AMPAR desensitization, another biophysical property altered by auxiliary subunit binding [11] [15] [22] [23], no difference was observed in cells expressing GluA2 alone or coexpressing GluA2 and SynDIG1 (Figure 1E).

Finally, it has been shown that CNIH, TARP, and GSGL1 auxiliary subunits alter the surface expression of AMPARs [12] [15] [18] [24]. To test if SynDIG1 regulates surface trafficking of AMPARs, S-AMPA was applied locally to the cell bodies of simultaneously recorded neighboring control and SynDIG1 shRNA expressing neurons in biolistically transfected slice cultures. The amplitude of the current in response to S-AMPA was indistinguishable in wildtype and transfected neurons, indicating that surface expression of AMPARs remains unchanged after loss of SynDIG1 (Figure 1F). As a positive control, we repeated this experiment with shRNA against the known AMPAR auxiliary subunit CNIH-2 instead of SynDIG1. Conditional knockout of CNIH-2 has been shown to reduce the current response to fast application of glutamate by 50%, as measured through outside-out patch recordings [18]. Using our local application method, we find a similar reduction in surface AMPARs, with CNIH-2 shRNA expressing neurons having an average current response that is 60% smaller than wildtype (Figure 1F). Taken together, the gating, pharmacological, and surface expression data indicates that SynDIG1 does not act as a typical AMPAR auxiliary subunit by modulating biophysical properties or trafficking of AMPARs.

### Overexpression of SynDIG1 Increases Excitatory Synaptic Transmission

**Figure 2:**
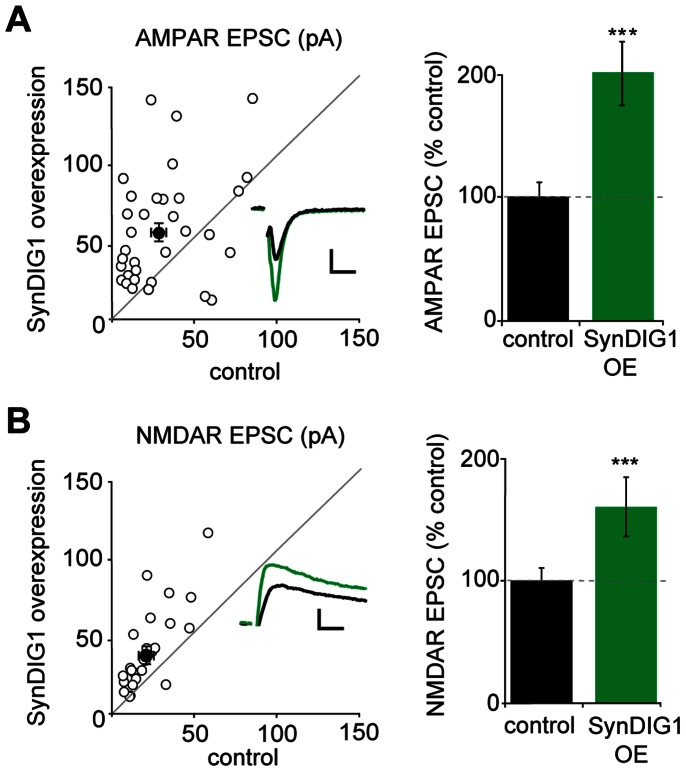
SynDIG1 overexpression increases both AMPAR and NMDAR EPSCs. (A+B) AMPAR- and NMDAR-mediated EPSCs recorded simultaneously from a neuron overexpressing SynDIG1 and a neighboring control neuron. Scatter plots show amplitudes of EPSCs for single pairs (open circles) and means ± SEM (filled circles). Inset are example traces for wildtype (black) and transfected (green) neurons. Scale bars represent 20 ms and 20 pA. Bar graphs (right) show average AMPAR and NMDAR EPSCs normalized to control (AMPA n = 33, *p = *0.0001; NMDA n = 25, *p* = 0.0009). Error bars denote SEM.

Having found that SynDIG1 does not act as an auxiliary subunit to AMPARs, we sought to better understand the effects of SynDIG1 on excitatory transmission by further characterizing the changes in synaptic transmission that occur in response to varying SynDIG1 levels. Previous research on SynDIG1’s role in synaptic transmission was carried out in dissociated hippocampal culture and only examined mEPSCs. We chose to more closely examine the effect of varying SynDIG1 levels using hippocampal slice cultures–a system that largely maintains the complex architecture of hippocampus, allowing for measurements of evoked transmission and the study of multiple features of synaptic transmission. Using biolistic transfection of hippocampal slice culture, we first overexpressed SynDIG1 in single hippocampal CA1 neurons and simultaneously recorded synaptic activity from transfected and neighboring control neurons in response to stimulation of Schaffer collaterals. We found that overexpression of SynDIG1 caused a doubling in AMPAR EPSCs compared to untransfected control neurons (Figure 2A). Surprisingly, in addition to the effect on AMPAR EPSCs, we also observed a significant increase in NMDAR-mediated EPSCs (Figure 2B).

### Knockdown of SynDIG1 Decreases Excitatory Synaptic Transmission

**Figure 3:**
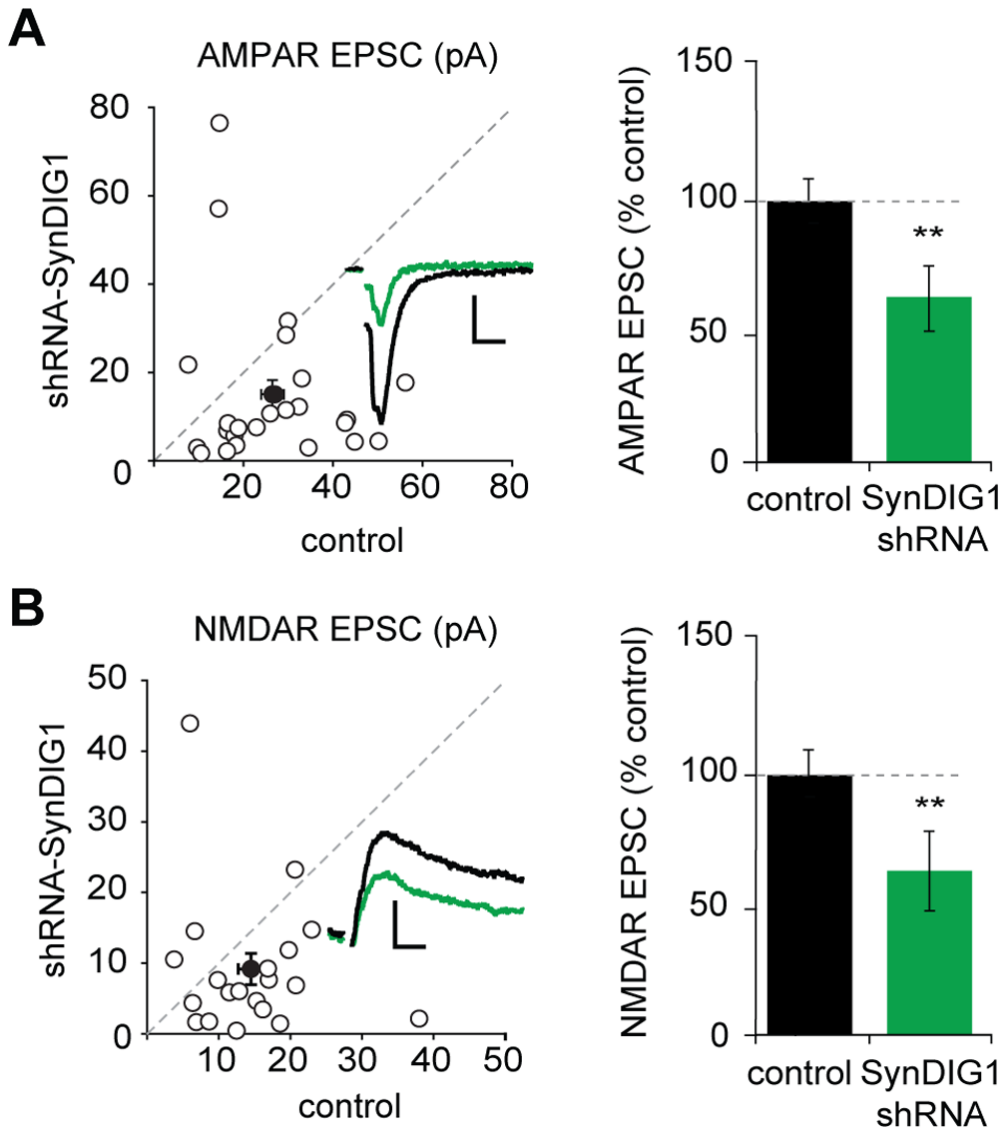
Knockdown of SynDIG1 results in a reduction in both AMPAR and NMDAR EPSCs. (A+B) AMPAR- and NMDAR-mediated EPSCs recorded simultaneously from a neuron expressing shRNA to knockdown SynDIG1 and a neighboring control neuron. Scatter plots show amplitudes of EPSCs for single pairs (open circles) and mean ± SEM (filled circles). Scale bars represent 20 ms and 10 pA. Bar graphs plotting average AMPAR and NMDAR EPSCs reveal a 40% decrease in transmission after knockdown (AMPA n = 25, *p* = 0.01; NMDA n = 20, *p* = 0.01). Error bars denote SEM.

If the increase in baseline synaptic transmission upon SynDIG1 overexpression reflects the endogenous role of the protein, reducing SynDIG1 expression should result in the opposite effect. To test this, we knocked down SynDIG1 using biolistic transfection of an shRNA that reduces SynDIG1 protein levels by 75% [17]. Simultaneous recordings of shRNA-expressing neurons and neighboring control neurons revealed a 40% decrease in both AMPAR-mediated (Figure 3A) and NMDAR-mediated EPSCs (Figure 3B), consistent with the conclusion that SynDIG1 expression levels regulate both AMPAR- and NMDAR-mediated transmission.

### SynDIG1 Expression does not Alter Presynaptic Release Probability

**Figure 4:**
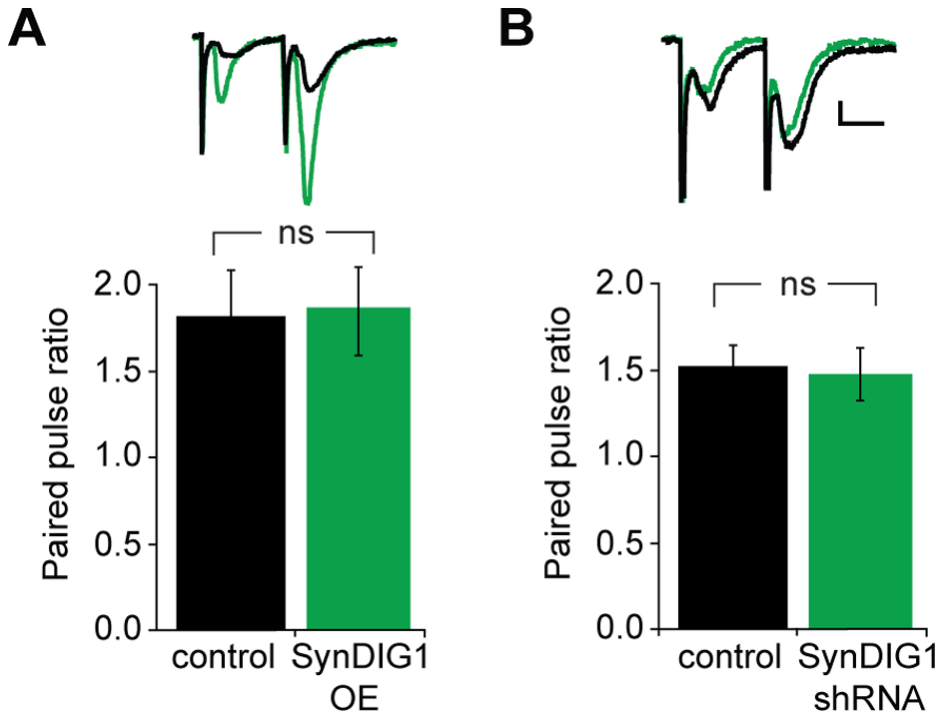
SynDIG1 expression does not alter probability of release. (A) Sample traces (above) and bar graph showing paired-pulse ratio mean ± SEM of control and SynDIG overexpressing cells. No change in paired pulse ratio was detected (n = 9, *p* = 0.69). (B) Sample traces (above) and bar graph showing paired-pulse ratio mean ± SEM of control and SynDIG shRNA-transfected cells. No change in paired pulse ratio was detected (n = 10, *p* = 0.71). Scale bars represent 15 ms and 10 pA.

A simultaneous change in both AMPAR- and NMDAR-mediated EPSCs can reflect a change in presynaptic transmitter release. To determine if the effect of SynDIG expression on excitatory transmission was driven by a change in presynaptic release probability, we compared the paired-pulse ratio of control neurons and neurons transfected with SynDIG1 or SynDIG1 shRNA. We did not detect a significant difference in the paired-pulse ratio between control and SynDIG1-overexpressing neurons (Figure 4A), nor did we observe a change in paired-pulse ratio after knockdown of SynDIG1 (Figure 4B), indicating that the change in EPSCs after manipulation of SynDIG1 expression reflects an change in total synapse number or postsynaptic strength, not a difference in presynaptic release properties.

### SynDIG1 Supports Excitatory Synaptogenesis

**Figure 5:**
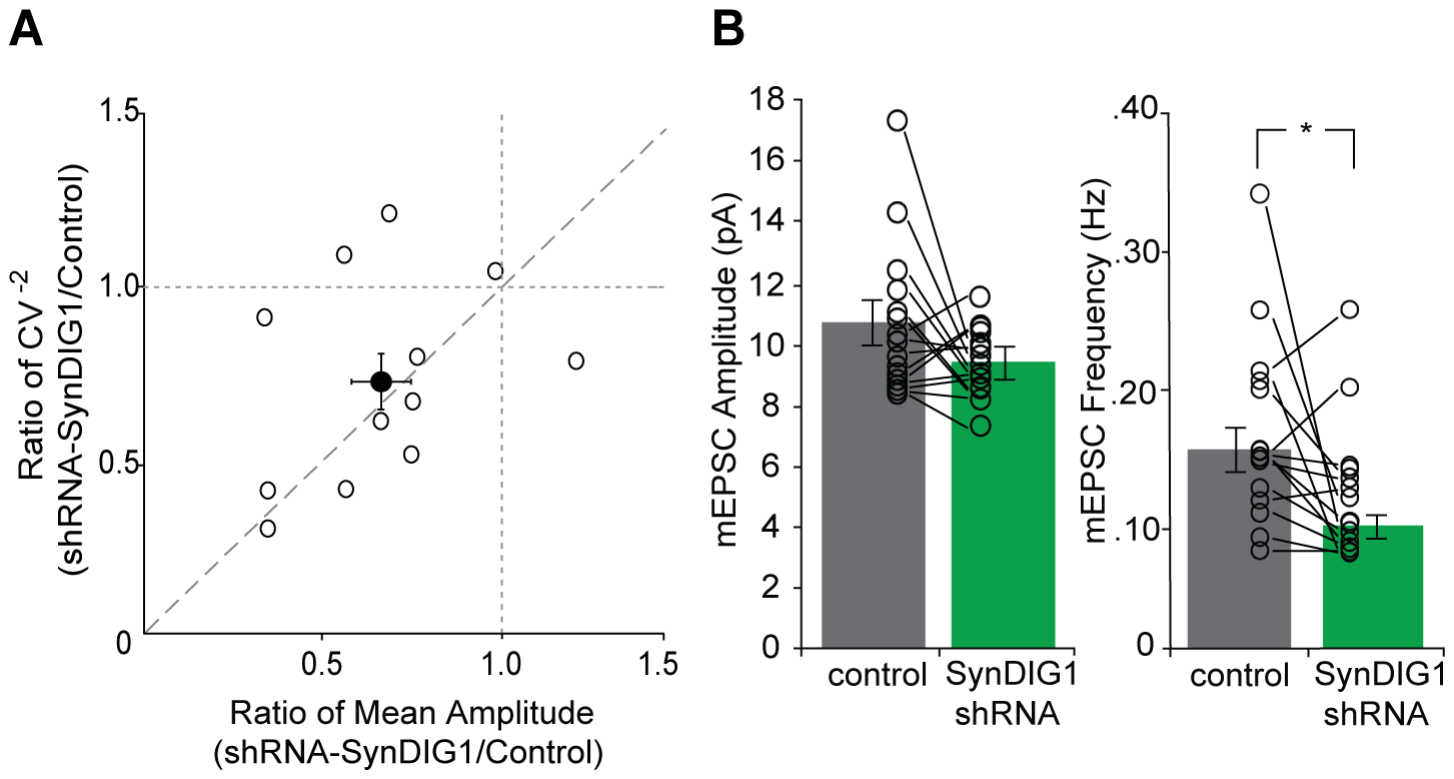
SynDIG1 regulates the number of functional excitatory synapses. (A) Coefficient of variation analysis for paired recordings of control and SynDIG1 shRNA expressing cells. Open circles represent pairs of neurons, filled circle represents mean ± SEM. Values on the y = x line indicate a change in the number of functional synapses, whereas values along the y = 1 line represent changes in synaptic strength of functional synapses. (B) Bar graphs depicting average mEPSC amplitude and frequency for control and SynDIG1 shRNA-expressing neurons. Reduction in SynDIG1 expression significantly reduces mEPSC frequency (n = 15, *p* = 0.04), but not amplitude (n = 15, *p* = 0.13). Error bars represent SEM.

Because SynDIG1 does not regulate presynaptic probability of release (Figure 4), the observed influence of SynDIG1 on synaptic transmission can either be due to a change in the number of synapses or the strength of existing synapses. To determine which of these two possibilities underlies the effects of SynDIG1 on EPSCs, we performed a coefficient of variation analysis of paired AMPAR EPSCs recorded from control cells and cells expressing SynDIG1 shRNA. In this analysis, a positive correlation between the ratio of transfected versus control coefficient of variation and the ratio of mean EPSC size indicates that fewer synapses are activated during smaller EPSCs, whereas no correlation indicates that fewer receptors per synapse are activated during smaller EPSCs [25]–[27]. We found that the shRNA-induced reduction in mean EPSC amplitude is correlated with a reduction in the ratio of the coefficient of variation (Figure 5A), suggesting that loss of SynDIG1 leads to a loss of excitatory synapses. To more directly quantify these changes, we also recorded mEPSCs after expression of SynDIG1 shRNA in hippocampal slice culture. Consistent with the coefficient of variation analysis, we observed a 40% decrease in the frequency of mEPSCs and no change in amplitude, supporting the conclusion that SynDIG1 regulates the number of excitatory synapses (Figure 5B).

## Discussion

All previously characterized AMPAR auxiliary subunits have been shown to regulate the biophysical properties of AMPARs [8] [10] [11] [15] [16] [22] [23]. Measuring kainate sensitivity, inward rectification, desensitization and deactivation rates, we found that SynDIG1 does not behave like known AMPAR auxiliary subunits by regulating receptor gating or pharmacology. Additionally, knockdown of SynDIG1 has no effect on whole-cell AMPAR currents, indicating that SynDIG1 does not play a role in surface trafficking of these receptors. Thus, it seems that despite its ability to bind directly to GluA2 in heterologous cells, SynDIG1 does not share the characteristics of a typical auxiliary subunit in hippocampal neurons.

To further investigate how SynDIG1 expression impacted excitatory synaptic transmission, we studied the effect of varying SynDIG levels in organotypic slice cultures. We found that SynDIG1 positively regulates both evoked AMPAR- and NMDAR-mediated currents; overexpression of SynDIG1 in neurons resulted in an increase in both AMPAR and NMDAR EPSCs, and knockdown of SynDIG1 decreased AMPAR and NMDAR EPSCs. While our AMPAR data is very much in agreement with the data published by Kalashnikova et al. [17], they did not observe a change in NMDAR mEPSCs following overexpression or knockdown of SynDIG1 in dissociated culture, which contrasts with our results on evoked NMDAR EPSCs. This difference could well be due to a difference in the sensitivity of the methodology; mEPSC recordings require a threshold for detection, which is made difficult with the slow NMDAR currents. Kalashnikova et al. [17] did observe an increase in NR1 puncta in response to SynDIG1 overexpression, indicating that SynDIG1 does alter NMDAR levels in dissociated neurons as well, though perhaps not to an extent that can be discerned in NMDAR mEPSC recordings from dissociated culture.

Because we found no difference in paired-pulse ratio, the observed change in synaptic AMPAR and NMDAR EPSCs can be attributed to a change in the number or strength of synapses. To determine whether SynDIG1 was necessary for synaptogenesis or synaptic strengthening, we performed a coefficient of variation analysis and mEPSC recordings in neurons expressing SynDIG1 shRNA. In agreement with previous findings [17], these experiments revealed a change in frequency of synaptic responses, consistent with a change in the number of synapses. However, Kalashnikova et al. also observed a significant decrease in mEPSC amplitude, though to a smaller extent than the change in frequency (50% decrease in amplitude versus 70% decrease in frequency) [17]. Again, this may be a result of the difference in our preparations, as dissociated cultures have larger mEPSC amplitudes on average, and the small decrease in amplitude we observed after SynDIG1 knockdown may have been larger and more pronounced in their system. Our coefficient of variation analysis, however, argues against this amplitude change contributing to the change seen with the evoked EPSC. Moreover, even in dissociated neurons, Kalashnikova et al. found a reduction in VGLUT1 puncta density after knockdown of SynDIG1, supporting our conclusion that loss of SynDIG1 leads to a loss of synapse number.

Recently, a number of groups have taken large-scale proteomics approaches to identifying AMPAR-interacting proteins [15] [16]. In each of these, SynDIG1 was not identified as a primary binding partner of AMPARs. Along with our data ruling out SynDIG1’s function as a traditional subunit, it is likely then that, despite its ability to bind AMPARs directly in a heterologous system, SynDIG1 may not interact with AMPARs directly in neurons, but instead acts as part of a larger postsynaptic complex that controls excitatory synapse formation. SynDIG1 has no known sequence homology with other postsynaptic proteins [8] [17], so we are not able to predict what type of protein-protein interactions SynDIG1 may form to regulate synaptogenesis. Interestingly, SynDIG4 (PRRT1) is thought to bind AMPARs directly *in vivo* [15] [16] and SynDIG1 was found to be capable of homodimerization [17], so it may be that SynDIG1 forms heterodimers with other SynDIG family members to exert its effects. Further research on binding partners of SynDIG1 will certainly prove useful in determining the specific mechanism by which SynDIG1 regulates excitatory synapse number.
